# Long non-coding RNA LINC00926 regulates WNT10B signaling pathway thereby altering inflammatory gene expression in PTSD

**DOI:** 10.1038/s41398-022-01971-5

**Published:** 2022-05-12

**Authors:** Marpe Bam, Xiaoming Yang, Jay P. Ginsberg, Allison E. Aiello, Monica Uddin, Sandro Galea, Prakash S. Nagarkatti, Mitzi Nagarkatti

**Affiliations:** 1 Department of Pathology, Microbiology and Immunology, School of Medicine, University of South Carolina, Columbia, SC 29209 USA; 2 Department of Epidemiology, UNC Gillings School of Global Public Health, University of North Carolina, Mcgavran-Greenberg Hall, Chapel Hill, NC 27599-7435 USA; 3 Genomics Program, University of South Florida College of Public Health, 3720 Spectrum Blvd., Tampa, FL USA; 4 School of Public Health, Boston University, 715 Albany Street - Talbot 301, Boston, MA 02118 USA

**Keywords:** Diseases, Genetics

## Abstract

Post-traumatic stress disorder (PTSD), which frequently occurs in the aftermath of a psychologically traumatic event is characterized by heightened inflammation. People with PTSD also suffer from a number of comorbid clinical and behavioral disorders that are related to chronic inflammation. Thus, understanding the mechanisms of enhanced inflammation in PTSD can provide insights into the relationship between PTSD and associated comorbid disorders. In the current study, we investigated the role of large intervening non-coding RNAs (lincRNAs) in the regulation of inflammation in people diagnosed with PTSD. We observed that WNT ligand, WNT10B, was upregulated in the peripheral blood mononuclear cells (PBMCs) of PTSD patients. This observation was associated with higher H3K4me3 signals around WNT10B promotor in PTSD patients compared to those without PTSD. Increased H3K4me3 resulted from LINC00926, which we found to be upregulated in the PTSD sample, bringing in histone methyltransferase, MLL1, onto WNT10B promotor leading to the introduction of H3K4 trimethylation. The addition of recombinant human WNT10B to pre-activated peripheral blood mononuclear cells (PBMCs) led to increased expression of inflammatory genes such as IFNG and IL17A, suggesting that WNT10B is involved in their upregulation. Together, our data suggested that LINC00926 interacts physically with MLL1 and thereby controls the expression of IFNG and IL17A. This is the first time a long non-coding RNA is shown to regulate the expression of WNT10B and consequently inflammation. This observation has high relevance to our understanding of disease mechanisms of PTSD and comorbidities associated with PTSD.

## Introduction

Large intervening non-coding RNAs (lincRNAs) are transcribed from thousands of loci in mammalian genomes and yet their precise role in human disease is not well understood. In the last decade or so, there has been growing interest in the mechanisms through which lincRNAs regulate gene expression. Some of the classic examples include (1) genomic relocalization of Polycomb complex (PRC2) and introduction of H3K27me3 to control cancer invasiveness by HOTAIR [1], function of XIST in silencing X chromosome in mammalian females for dosage compensation [2] and the like. Similarly, several genes related to immune system have also been reported to be regulated by lincRNAs. To mention a few, lincRNA, TMEVPG1, was shown to positively regulate the expression of IFNG in Th1 cells [3]. As another example, it was shown that lincRNA-Cox2 positively regulates the expression of IL12B gene through the introduction of H3K27me2 [4]. Recently, our laboratory reported that lncRNA AW112010 enhances the differentiation of inflammatory T cells by suppressing IL-10 expression [5]. Thus, the role of lincRNA in the context to immune system gene regulation is slowly beginning to emerge [6].

The WNT signaling pathways are a group of signal transduction pathways that play a critical role in early development and later during growth as well as maintenance of homeostasis of various tissues. Signaling starts by binding of the WNT ligand to its receptor on the cell surface. There are 19 known WNT ligands. All these ligands trigger similar signaling mechanisms but each one of them is involved in different cell types. More recently, it has been reported that WNT signaling pathway may be involved in the regulation of immune system genes including inflammatory genes to contribute to disease pathophysiology [7].

Posttraumatic Stress Disorder (PTSD) is a psychiatric disorder with incompletely understood pathophysiology. Exposure to a psychologically traumatic event puts some persons at risk of developing PTSD [8–10]. PTSD occurs among persons who experience violence and trauma like domestic abuse, war, accident, sudden death of loved ones, and the like. The prevalence of PTSD can reach as high as 40% among those who experienced severe traumatic events like rape and up to 30% among war veterans compared to about 3.6% among American adults [11–13]. Additionally, in patients who were hospitalized due to COVID-19 and recovered, the prevalence of PTSD can be up to 30% [14]. In the Detroit Neighborhood Health Study, the prevalence of PTSD has been reported to be more than 17% [15].

In our previous studies, we noted that PTSD was associated with heightened inflammatory phenotype with increased induction of pro-inflammatory cytokines such as IFNG and IL17 [16–18]. Our studies demonstrated that epigenetic modulations impacting the inflammatory cytokine genes may play a crucial role in their induction. PTSD patients have a higher risk of developing a variety of other disorders including asthma, chronic obstructive pulmonary disease, chronic fatigue syndrome, arthritis, fibromyalgia, migraine headaches, cancer, and other respiratory, cardiovascular, gastrointestinal, or pain disorders [19, 20], the majority of which have an inflammatory component. Thus, studies aimed at better understanding the inflammatory pathways in PTSD may help illuminate the pathways that explain the link between several comorbidities with PTSD.

It is worth mentioning that the involvement of lincRNA-regulated WNT signaling in the inflammatory state has not been reported before in patients with PTSD. Thus, this study aimed to examine regulation of inflammatory gene expression by epigenetic mechanisms. We studied the role of lincRNA, LINC00926, in the regulation of WNT10B to understand the regulation of inflammatory cytokine genes in PTSD. Our studies demonstrate that inflammatory gene expression associated with PTSD is regulated by LINC00926 by bringing MLL1 to the promotor of WNT10B to induce H3K4me3, resulting in its elevated expression. Our report is the first to show the involvement of lincRNA in regulating WNT signaling that influences inflammation in PTSD.

## Methods

### Patients

We provided informed and written consent to all participants and the study was approved by the University of South Carolina Institutional Review Board. We included 48 samples (equal number of controls and PTSD) in total. The samples were obtained from two studies. One group consisted of Veterans of either the Persian Gulf War (1991) or the Operation Enduring Freedom and Operation Iraqi Freedom (OEF& OIF) campaigns. The demographic data were reported previously [16], and the other group included study participants from the Detroit Neighborhood Health Study (DNHS) [15]. All donors of the first study group (war veterans) were clinically assessed by professionals for PTSD employing the psychometric properties of the PTSD Checklist (PCL) [21] and the PTSD diagnosis was further validated by the gold-standard Clinician Administered PTSD Scale according to formal diagnostic criteria in the Diagnostic and Statistical Manual of Mental Disorder (DSM-IV) [22]. For DNHS samples, PTSD was diagnosed using structured telephone interviews and the PTSD Checklist-Civilian Version (PCL-C) followed by in-person clinical interviews using the Clinician Administered PTSD Scale as reported previously [15]. Exclusion criteria included current alcohol and other substance abuse, cancer, undergoing immunosuppressive drug treatment or having immunosuppressive disease. Patients with active infections during the time of PBMC collection were also excluded. The control individuals were age-matched healthy volunteers, who did not have any symptoms of active infection, psychiatric disorder, or any history of immune compromise such as HIV, cancer, pregnancy, or on chronic steroid therapy.

### Sample collection and RNA isolation

Peripheral blood samples (2–10 ml) collected in EDTA coated collection tubes were processed to isolate PBMCs by density centrifugation using Ficoll-Paque (GE Healthcare, Uppsala, Sweden). Using a universal kit (AllPrep DNA/RNA/miRNA Universal Kit, Qiagen, Valencia, CA) recommended for simultaneous isolation of high quality DNA and total RNA, including miRNAs, all the three entities were isolated from the same PBMCs and immediately frozen at −80 °C until use.

### RNA-sequencing (RNA-seq)

Five control and 5 PTSD patient samples from Dataset 1 were analyzed. Libraries were constructed using Illumina TruSeq RNA Sample Preparation kit as described in Bam et al., (2016). Briefly, total RNA from PBMCs was isolated using the Qiagen AllPrep DNA/RNA/miRNA Universal Kit. Following the manufacturer’s instruction, oligo-dT beads were then added to 1 µg of total RNA to isolate mRNA. The mRNA obtained was fragmented to 200–400 bases. The RNA fragments were then reverse transcribed into double stranded cDNA fragments followed by repairing the DNA fragments to generate blunt ends using T4 DNA polymerase, Klenow polymerase, and T4 polynucleotide kinase. The DNA fragments were purified using Qiagen PCR purification kit (Qiagen #28004), following which an “A” base was added to the 3’ end of the blunt DNA fragment by Klenow fragment. Using DNA ligase, sequencing adapters were ligated to the ends of DNA fragments. The libraries were then amplified by limited PCR cycles (15 cycles) using primers provided in the kit. The PCR products were gel separated by running in a 2% agarose gel and fragments with sizes ranging from 250 to 400 bp were excised and purified using the QiAquick Gel Extraction Kit (Qiagen #28704). The concentration of the libraries was determined by a NanoDrop spectrophotometer (Thermo Scientific, Wilmington, DE). The library was sequenced by Illumina HiSeq 2000 at Tufts University Genomic core facility.

### RNA-seq data analysis

Single-end sequencing generated about 20 million reads for each sample. Reads were mapped to human genome build hg19 using Tophat 2 with default setting allowing 2 final read mismatches [23]. The mapped reads were then filtered and uniquely mapped reads in SAM format were used for transcript assembling and Fragments Per Kilobase of transcript per Million mapped reads(FPKM) calculation using Cufflinks [24]. The differentially expressed transcripts were determined by Cuffdiff and Cuffcompare with false discovery rate set as 0.05 [24].

### RNA–protein interaction prediction

Two freely available online tools, lncPro (http://bioinfo.bjmu.edu.cn/lncpro/) and RPISeq (http://pridb.gdcb.iastate.edu/RPISeq/) [25, 26] were used to predict the interaction between LINC00926 and MLL1/KMT2A. We included HOTAIR and EZH2 as the reference since these molecules were previously shown to interact with each other to bring about histone modifications around its target gene [1]. To run the tools, we simply followed the instructions provided and obtained the interaction scores generated by the tools using default setting. RPISeq provides two scores as RF classifier and SVM classifier and lncPro provides one value.

### Cell culture

THP1 cells obtained from ATCC were cultured in complete RPMI medium containing 10% FBS, penicillin and streptomycin, HEPES buffer, and 2-mercaptoethanol. TALL-104 cells, also from ATCC, were cultured in medium containing the required ingredients as per the recommendations by the ATCC. The incubation condition was at 37 °C and 5% CO2.

### WNT10B signaling pathway initiation

To understand whether WNT10B influenced the expression of proinflammatory genes, WNT signaling was promoted by adding recombinant human (rh)-WNT10B in PBMC cultures. The PBMCs were isolated from healthy human individuals. Two million cells were cultured in 200 µl complete RPMI medium in a 96 well tissue culture plate as follows: PBMCs were first stimulated with Phorbol 12-myristate 13-acetate (PMA) (200 nM) for 6 h following which, 200 ng/ml rh-WNT10B (Cat# 7196-WN-010/CF, R&D Systems) was added to treatment groups. PBMCs were harvested after 24 or 48 h and used for RNA and whole cell lysate (WCL) extraction for further analysis. Culture supernatants were also collected for ELISA to detect IFNG. The following combinations were used as controls: 1. PBMCs + PMA without WNT10B; 2. PBMCs + WNT10B without PMA.

### Blocking WNT signaling

To investigate whether WNT10B was directly responsible for the upregulation of IFNG expression, we blocked the WNT signaling pathway by using ICG-001 (10 µM), a known inhibitor of WNT signaling pathway [27]. PBMCs (2 × 10^6^) from healthy controls were cultured overnight (~18 h) with 10 µM of ICG-001 in separate wells of a 96 well culture plate in 200 µl complete RPMI medium. The following day, PMA + Ionomycin and ICG-001 (in a 10 µl volume) were added to the culture to reach final concentrations of 200 nM PMA and 10 µM ICG-001, respectively. After 6 h, WNT10B (200 ng/ml) was added to appropriate wells. After 24 and 48 h of addition of WNT10B, cells were harvested for total RNA extraction. Culture supernatant was collected for ELISA to detect IFNG.

### In vitro knockdown with siRNA

To analyze whether the presence of H3K4me3 influenced the expression of WNT10B, we knocked down MLL1 [28] (Lysine Methyl Transferase 2 or KMT2A etc.) and KDM5B [29] (Lysine (K)-specific demethylase 5B) in separate experiments and quantified WNT10B transcripts. For the knockdown of MLL1, PBMCs from a healthy donor were plated at 5 × 10^5^ cells per well in a 24-well plate. After the cells had settled at the bottom of the well (about 1 h), 5pmole of siRNA was added as a micelle mixture in Lipofectamine RNAiMAX^®^ (Invitrogen, ThermoFisher Scientific, USA) to each well by following the instruction from the manufacturer. For the KDM5B knockdown assays, THP1 cells were plated at 0.2 million per well a day before the transfection. Similarly, for knock down experiments using TALL-104, protocol followed for THP1 was employed. The cells were further cultured for the indicated time and harvested for RNA isolation and later used for qRT-PCR analysis.

### ELISA

ELISA to detect human IFNG was performed on culture supernatants with ELISA kit purchased from Biolegend Inc. (CA, USA) and assay was performed by following the manufacturer instructions. Reading for OD was performed in a Victor^®^_TM_, 1420 Multilabel Counter (Perkin Elmer, USA) plate reader.

### ChIP-qPCR

Approximately 10 million PBMCs from a healthy donor were fixed and crosslinked with methanol-free formaldehyde (1% final concentration) for 15 min. Remaining of the protocol was the same as described earlier for ChIP-seq sample preparation. IP was performed using anti-MLL1 antibody from Active Motiff (cat#61295). The RNA obtained after ChIP was used for the qPCR quantification of the LINC00926 after converting the RNA into cDNA. All reagents used were RNase-free. The sequence of the primer used for qPCR is provided in Table 1.

**Table 1:** Sequences of the primers used for the ChIP-qRT-PCR.

*Primer name*	*Primer sequence*
*Wnt10Ba f*	*5*′*GGG GGC ACT CCT TTA TTC TC*
*Wnt10Ba r*	*5*′ *ACC CAA ACC ACT GGA GGT AA*
*Wnt10Bb f*	*5*′ *CTT AAA CCG TGG GGA GAC TG*
*Wnt10Bb r*	*5*′*GCT TGC TAG CTC TCT CGA TCA*
*Wnt10Bc f*	*5*′ *CTC CAC TCC CAG CCT TGA C*
*Wnt10Bc r*	*5’* *ACT CTG GCC ACC TCC TTC TT*
*Wnt10Bd f*	*5*′ *CTC CAC TCC CAG CCT TGA C*
*Wnt10Bd r*	*5*′ *ACT CTG GCC ACC TCC TTC TT*
*Wnt10Be f*	*5*′ *ACC TTC CTA GGG AGC CAG AA*
*Wnt10Be r*	*5*′ *GAC TGT CCC ACA CCG AGA AG*
*Wnt10Bf f*	*5*′ *CTT GGT TCC CAC CCT CCT*
*Wnt10Bf r*	*5*′ *GGA TAC CTC TGT TGG CAA GC*
*Wnt10Bg f*	*5*′ *CTT GGG ATC CAA AAT TGA GC*
*Wnt10Bg r*	*5*′ *GAG CTT CGA GGG TGA GTC AG*
*Wnt10Bh f*	*5*′ *AAG CAG AGG AAC AGG GAT GA*
*Wnt10Bh r*	*5*′*GAC CTC CAG CAG CAC AGA CT*
*Wnt10Bi f*	*5*′ *TGA GAC AGG AGA TCG AGC AA*
*Wnt10Bi r*	*5*′ *GGA AGG AGG GTC AGG AAC TC*
*Wnt10Bj f*	*5*′ *GGA CAA GAG CCA AAC CAT GT*
*Wnt10Bj r*	*5*′ *GAG AGG CTT AGG CAA CCT CA*
*Wnt10Bk f*	*5*′ *CCC CCT TAG GTT TGG GAT TA*
*Wnt10Bk r*	*5*′ *CCC AGA GTT GGG CTT CAG TA*
*Wnt10Bl f*	*5*′ *CAG ACT GGG TGG CCT GAG*
*Wnt10Bl r*	*5*′ *GAC CCA CGT CAA GGT CTC TC*
*Wnt10Bm f*	*5*′ *AGG GAG GGA CTA GGA GCA AG*
*Wnt10Bm r*	*5*′ *GAC ATC CCC CAA TCT CCA G*
*Wnt10Bn f*	*5*′ *GGG TGA CTC ATC TGT TGC TG*
*Wnt10Bn r*	*5*′ *CTT TGG TGC CTC CAG GTG*
*Wnt10Bo f*	*5*′ *AGA ATT TTG CCC CCT TCT GT*
*Wnt10Bo r*	*5*′ *TTG CCT TGA CTT TGG GAT CT*

Approximately 3 kb (2 kb upstream and 1 kb downstream of TSS) of the human Wnt10B promotor region was covered by 15 sets of primers. Each primer set amplified a region of ~200 bp such that the whole ~3 kb fragment was completely covered. *f* forward primer, *r* reverse primer.

For the detection of the WNT10B promotor and MLL1 complex, ChIP was performed in the same way as described above except that the DNA obtained at the end was directly used for PCR with the primers detailed in Table 1. Fifteen primer sets covering a span of 3 kb on the WNT promotor region (2 kb upstream and 1 kb downstream from TSS) were used to perform the PCR with the IP DNA samples to detect the genomic region. Each primer pair covered about 200 bp of nucleotides.

### Statistical analyses

PTSD and normal control samples were randomly selected for RNA-seq and real-time qPCR experiments. The heatmap of RNA-seq result was generated using R package. The *p* values for multiple comparisons were calculated by the t-test adjusted with Benjamini & Hochber (BH) method. For statistical comparison between the two groups, the mean difference between controls and PTSD patients were screened by using an unpaired, two-tailed standard t-test and *p* < 0.05 was considered to show a significant difference. The data are presented as mean ± standard deviation. For in vitro cell culture experiments, each experiment was repeated at least three times with consistent results.

## Results

### LINC00926 is upregulated in PTSD PBMCs

**Fig. 1:**
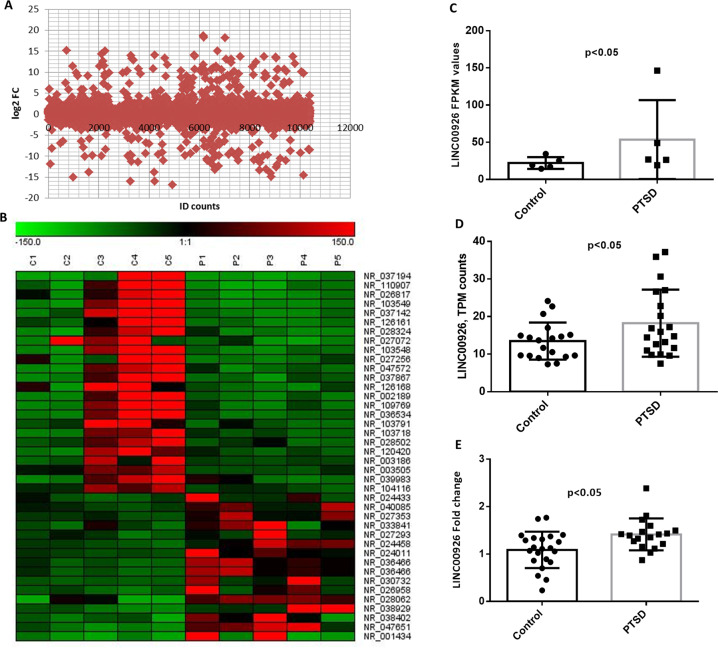
Expression of noncoding RNAs in the PBMCs of PTSD patients. RNAseq was performed on total RNA from controls and patients. Only noncoding RNA differential expression was analyzed. **A** Scattered plot showing all the noncoding RNAs with at least 1 FPKM after RNAseq analysis. **B** Heatmap of only significantly dysregulated lncRNAs in PTSD. The labels on the right side indicate the NCBI accession numbers of the corresponding lncRNA. We then focused on the expression of LINC00926 for our further studies. **C** Expression of LINC00926 in PTSD patients’ PBMCs from our RNAseq analysis. *P* value was obtained from the tool to analyze the RNAseq data. **D** RNAseq data from GSE114407 was used to analyze the expression of LINC00926 in PTSD patients PBMCs and the figure depicts the TPM values obtained from the dataset where the *p* value was obtained after performing Student’ *t* test. Then we validated the expression of LINC00926 in PBMCs by performing qRT-PCR **E**. (FPKM: Fragments Per Kilobase of transcripts per Million mapped reads; TPM: Transcripts per million; Each dot in the plots represents an individual sample included for the analysis).

Recently, we reported that WNT10B promotor had higher H3K4me3 signals in PTSD patients when compared to normal individuals [30]. Consequently, we hypothesized that this may result from co-operative function of LINC00926 and MLL1/KMT2A where, LINC00926 helps in bringing MLL1 to the promotor of WNT10B resulting in increased H3K4me3. Thus, we first analyzed the expression of LINC00926 in PTSD PBMCs by RNA-seq as well as mining data from publicly available datasets. As seen in Fig. 1, we found several differentially expressed non-coding RNAs in PTSD (Fig. 1A) and 40 of them, including LINC00926 (Fig. 1B, C, respectively), had significant differential expression. We also checked the expression of LINC00926 in the GEO dataset GSE114407 (RNAseq data from PBMCs from PTSD and controls from a different study) and observed that LINC00926 was significantly upregulated (Fig. 1D). Next, we validated the expression of LINC00926 by qRT-PCR after including more samples and observed a similar pattern (Fig. 1E) as seen in the sequencing data.

### LINC00926 interacts with histone methyltransferase MLL1 and regulates the expression of WNT10B

**Fig. 2:**
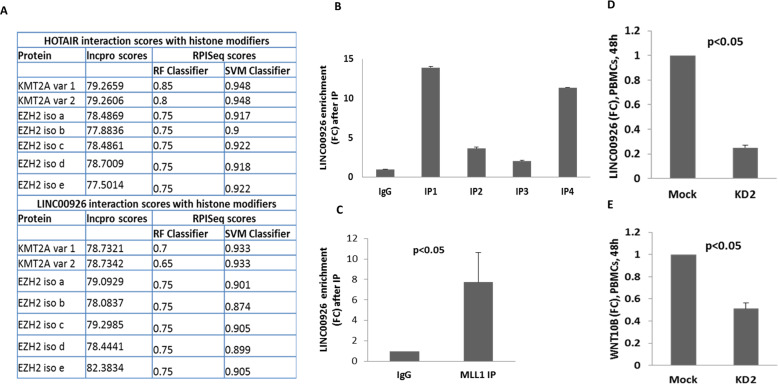
LINC00926-MLL1 interaction in order to regulate WNT10B expression. We predicted that LINC00926 interacted physically with MLL1 to introduce H3K4me3 around WNT10B promotor. Thus, we first analyzed the RNA–protein interaction by online prediction tools, performed ChIP-qPCR and siRNA-based knockdown to see the effect on WNT10B expression. **A** provides the predicted interaction scores for LINC00926-MLL1 and HOTAIR-EZH2 from the analysis tools. Following this, four separate co-immunoprecipitation using Ab against MLL1 to pull down LINC00926 and qRT-PCR to detect its enrichment was performed on whole cell extract from fixed and crosslinked PBMCs from healthy control to show that these two molecules are physically associated (**B**). **C** provides the mean of the four co-immunoprecipitations. Then, in order to show that LINC00926 influenced the expression of WNT10B, we knocked down LINC00926 in PBMCs by employing siRNA and measured the WNT10B transcripts by qRT-PCR (**D**, **E**). Each dot represents a human participant.

In order to understand whether LINC00926 could possibly interact with MLL1, we first checked the RNA–protein interaction prediction scores. For the purpose of comparison, we included HOTAIR and EZH2 interaction as the reference point since their interaction was previously reported in the context of gene regulation [31]. The scores from two different prediction tools indicated that the interaction between LINC00926 and MLL1/KMT2A is highly likely as the scores for HOTAIR and EZH2 were very close to that of the molecules in our study (Fig. 2A). Moving further, to understand the regulation of WNT10B expression considering higher H3K4me3 levels around its promotor in PTSD patients [30], we hypothesized that LINC00926 brings methyltransferase MLL1 to the WNT10B promotor through physical interaction resulting in the incorporation of H3K4me3 around the WNT10B promotor. This hypothesis was based on 3 rationales: (1) our observation of increased LINC00926 in PTSD, (2) high scores for strong protein–RNA interaction between LINC00926 and human MLL1 (KMT2A) and 3) the fact that LINC RNA physically interact with different proteins to regulate gene expression [1–4]. Thus, as seen in Fig. 1C–E, we noted a significant increase in LINC00926 in PTSD patients. Next, to prove that LINC00926 was actually interacting with MLL1 to form a complex, we performed ChIP assay with antibody against MLL1 and quantified the enrichment of LINC00926 by qPCR. Using this assay, we observed that LINC00926 was several fold enriched (Fig. 2B, C) in the ChIP samples indicating that LINC00926 and MLL1 physically interacted to form a complex. Further, to prove that LINC00926 regulated the expression of WNT10B, we knocked down LINC00926 with siRNA and quantified the transcripts of WNT10B. We observed that upon knocking down of LINC00926, the transcript level of WNT10B was found to be downregulated (Fig. 2D, E).

### MLL1 and LINC00926 are physically associated with the promotor of WNT10B

**Fig. 3:**
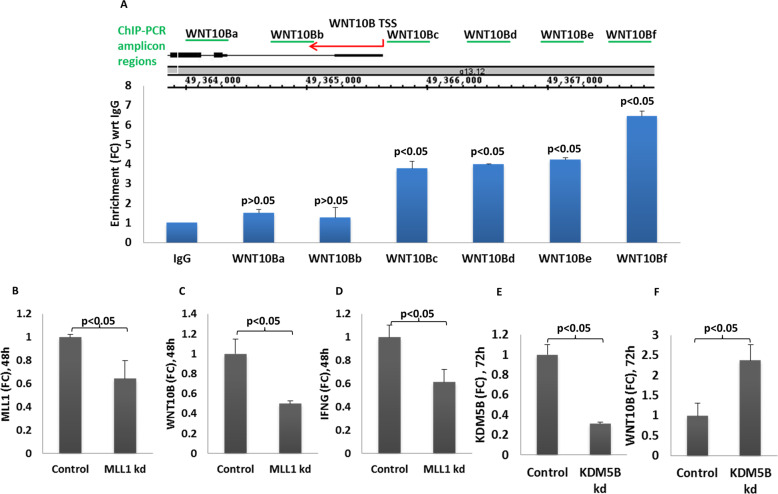
WNT10B promotor engagement by MLL1, via LINC00926, plays a role in its expression. ChIP-qPCR was performed on DNA samples obtained after co-IP with anti-human MLL1 on whole cell lysate from fixed and crosslinked PBMCs. Fifteen primer pairs which covered ~2 Kb upstream and ~1Kb downstream from WNT10B transcription start site were used for qPCR to detect enrichment of the DNA after co-IP. In another experiment, either MLL1 or KDM5B was knocked down with siRNA to see if it affected the expression of WNT10B and hence IFNG. Of the 15 primer sets only 4 (WNT10Bc-f) primers showed enrichment of the WNT10B promotor region (**A**). Histone writer (MLL1) and eraser (KDM5B) were knocked down with siRNA and its effect on the expression of WNT10B and IFNG shown as measured by qRT-PCR. **B**–**D** Similarly, the effect of reduction in KDM5B on WNT10B in THP1 cells is shown, as measured by qRT-PCR (**E**, **F**).

We next tested to see if MLL1, LINC00926, and the WNT10B promotor region were in a complex, physically interacting with each other, to achieve regulation of WNT10B expression. To that end, we performed ChIP-qPCR on DNA obtained from ChIP samples using human Anti-MLL1 to perform IP. We used 15 primer sets to cover 3 kb of the WNT10B promotor region. As seen in Fig. 3A, only WNT10Bc, d, e, and f primers showed significant enrichment compared to IgG. These primers had binding sites on the upstream of transcription start site (TSS) on WNT10B. However, the remaining 11 primers (WNT10Ba, b, c—as shown in Fig. 3A and 8 more primers, which are not shown in Fig. 3A), covering mostly downstream of WNT10B TSS, did not show any enrichment after ChIP-qPCR. This result corroborated well with our ChIP-seq data (Fig. 1B) wherein we observed H3K4me3 peaks mostly near or in the upstream of TSS of WNT10B promotor. Furthermore, as shown previously in Fig. 2C, D, it is clear that MLL1 is in contact with LINC00926 as well. Thus, together these observations suggested that MLL1, LINC00926, and WNT10B promotor are in physical contact with each other to form a complex in the PBMCs.

### Alteration in methylation writer or eraser level affects WNT10B expression

MLL1 is an H3K4me3 methyltransferase (writer) and KDM5B is an eraser of methyl groups. Thus, we knocked down these two enzymes in separate experiments and quantified WNT10B and also IFNG transcripts. When we knocked down MLL1, we observed that WNT10B transcript also reduced effectively (Fig. 3B, C). Furthermore, we also observed reduced IFNG transcript in the same samples (Fig. 3D). In contrast, knocking down of KDM5B resulted in increased transcript level of WNT10B (Fig. 3E, F), indicating that histone methylation was involved in regulating WNT10B expression.

### Increase in WNT10B promotes proinflammatory cytokine gene expression

**Fig. 4:**
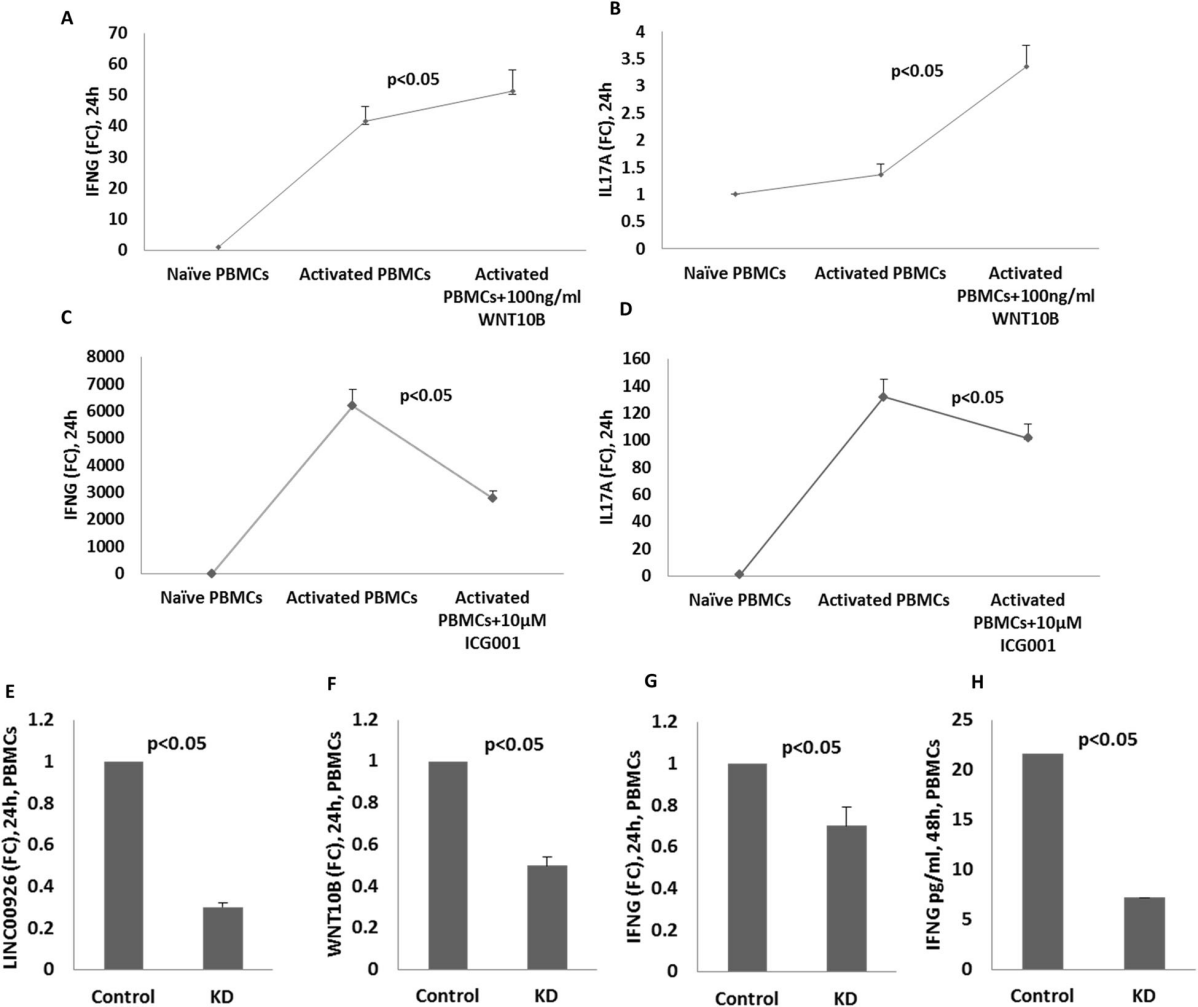
Regulation of inflammatory gene expression by WNT10B. In order to evaluate the effect of increased WNT10B on the expression of inflammatory genes, pre-activated PBMCs were exposed to recombinant human WNT10B from outside. Levels of IFNG and IL17A transcripts were then measured after the indicated duration of culture. In another experiment, inhibitor (ICG001) of WNT signaling pathway was used to see whether WNT signaling was responsible for the increased expression of IFNG and IL17A. Next, in order to show that LINC00926 was responsible for the increased expression of IFNG, LINC00926 was knocked down in PBMCs and the levels of WNT10B and IFNG was measured by qRT-PCR. Effect of enhanced WNT10B signaling on IFNG and IL17A in pre-stimulated PBMCs (**A**, **B** respectively) and upon inhibition of WNT signaling, on IFNG and IL17A as measured by qRT-PCR (**C**, **D** respectively). Effect of LINC00926 knock down on WNT10B and IFNG expression as measured by qRT-PCR in PBMCs (**E**–**G**) and by ELISA of culture supernatant for IFNG (**H**).

Next, we tried to test if WNT10B was responsible for the upregulated expression of IFNG and IL17A in PTSD, and consequently, mimicked the upregulated level of WNT10B by adding recombinant human WNT10B into the culture medium consisting of pre-activated PBMCs from healthy donors and compared the level of IFNG and IL17A. The addition of WNT10B increased the level of IFNG and IL17A in activated PBMCs (Fig. 4A, B). To confirm that indeed WNT signaling (WNT10B in this case) was responsible for the upregulation of IFNG and IL17A, we also blocked WNT signaling by adding ICG-001, a small molecule that inhibits canonical WNT pathway [27]. The concentration of the inhibitor used in this experiment was based other publications as well as our previous experiments [30, 32, 33]. Indeed, the addition of the WNT signaling inhibitor significantly reduced the level of IFNG and IL17A in activated PBMCs (Fig. 4C, D).

### Reduction in LINC00926 results in reduced IFNG

**Fig. 5:**
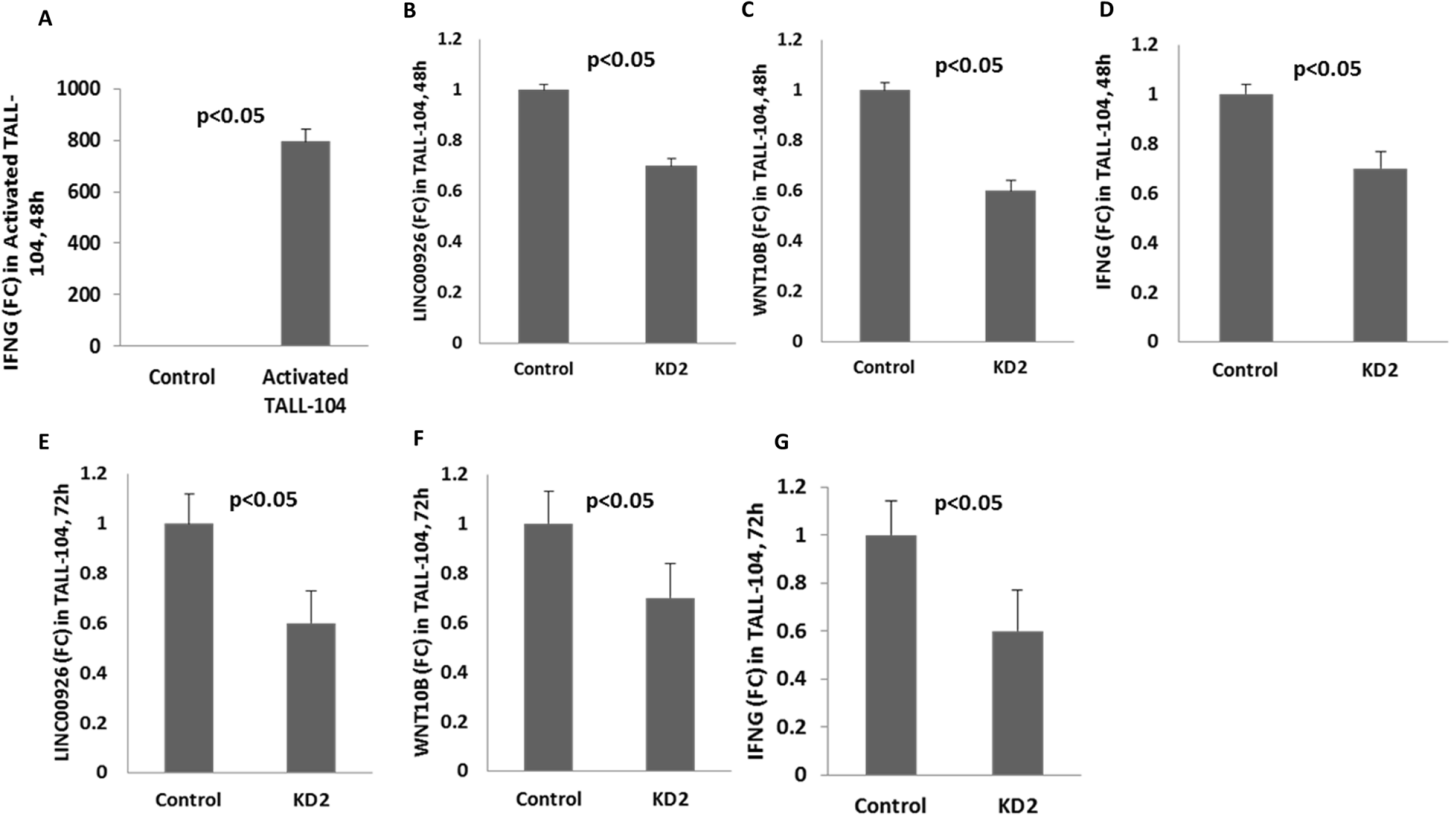
Regulation of IFNG expression by WNT10B in TALL-104 cells. To confirm that WNT10B regulated IFNG as a result of the role played by LINC00926 in T cells, TALL-104, a human lymphoblastic cell line known to express IFNG was used. LINC00926 was knocked down in these cells using siRNA and WNT10B and IFNG were measured by qRT-PCR at the indicated time points. Induction of IFNG in activated TALL-104 (**A**). Effect of knocking down of LINC00926 (**B** 48 h, and **E** 72 h) on WNT10B at different times (**C** 48 h and **F** 72 h) and on IFNG levels (**D** 48 h and **G** 72 h), as measured by qRT-PCR. KD2: 2nd Knockdown, representing the siRNA used.

To confirm that LINC00926 was responsible for the upregulated expression of WNT10B and consequently the elevated expression of inflammatory genes, we knocked down LINC00926 by employing siRNA and analyzed the level of WNT10B and IFNG. We performed the knock down experiments in different cell types including PBMCs and TALL-104, a lymphoblastic cell line known to express IFNG. We observed that lowering the level of LINC00926 also led to decreased levels of WNT10B expression. This observation was consistent in all the experiments that we performed using PBMCs (Fig. 4E–H) and TALL-104 (Fig. 5). Furthermore, as seen in the same figures (Figs. 4 and 5) the level of IFNG also decreased significantly upon knockdown of LINC00926. These data suggested that LINC00926 was involved in the regulation of WNT10B expression and this eventually contributes to the upregulated expression of inflammatory genes.

## Discussion

**Fig. 6:**
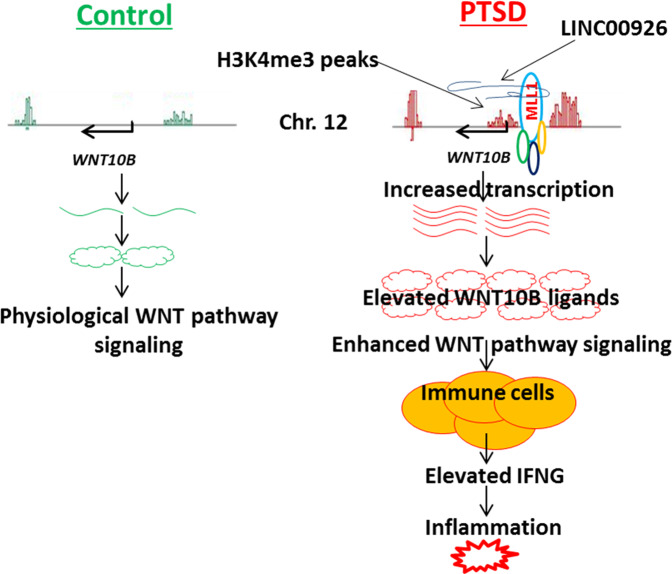
Schematic explaining the role of LINC00926 in orchestrating the regulation of WNT10B in order to regulate IFNG expression. In order to introduce H3K4me3 methylation around WNT10B promotor, LINC00926 associates with MLL1 and brings it to the promotor of WNT10B. This presence of MLL1/LINC00926 complex around WNT10B promotor upstream of TSS, introduces H3K4me3 in this region in PTSD. This results in easy access of the transcription complex and thereby increased transcription of WNT10B leading to increased WNT signaling in PTSD. Consequently, the expression of inflammatory genes like IFNG and IL17A are elevated due to increased WNT signaling, leading to inflammatory state in PTSD patients. On the contrary, in controls, WNT10B expression is not altered and therefore it is present at physiologically favorable levels, and therefore, the expression of inflammatory genes is not affected.

Although PTSD is considered as a neurological disorder, it is well known that PTSD is associated with a number of comorbidities that involve an inflammatory state, such as the metabolic syndrome, atherosclerotic cardiovascular disease, and autoimmune diseases [34]. Despite extensive research on PTSD, little progress has been made in the mechanisms that trigger the inflammatory state in PTSD Fig. 6. PBMCs are critical for proper immune response, and uncontrolled inflammation can damage neurological tissues. It is known that increased amount of PBMC and altered gene expression in PBMC are associated with the onset and severity of PTSD symptoms [35]. On the other hand, stress could affect the function and gene expression in PBMCs [36]. Although it is unclear how PBMCs and CNS interact with each other, studies in animal models have shown that activated PBMCs can cause inflammation in CNS tissue [37], while Treg cells can reduce anxiety symptoms [38]. Therefore, the damage to the CNS tissue in PTSD could be related to the inflammation caused by infiltration of inflammatory PMBCs as well as cytokines produced by PBMCs. In fact, in an earlier study, we reported that PTSD patients had increased levels of PBMC-derived inflammatory Th1 and Th17 cells which correlated positively with the PTSD scores [16].

Epigenetic regulation plays very important roles in gene expression in response to environmental signals including stress and trauma. For example, histone/DNA modifications and miRNAs are known to regulate the genes related to PTSD development and other comorbidities associated with PTSD [39–43]. A genome-wide DNA methylation study in PBMCs has identified genes whose DNA methylation levels are associated with PTSD [44]. In animal models, alteration in histone acetylation is associated with fear extinction and learning [45, 46]. Many of those genes with altered histone/DNA modification in PTSD are related to immune response. We and others have shown epigenetic regulation in the expression of inflammatory cytokine genes in PBMCs from PTSD patients [16–18, 47]. Recently, an emerging body of evidence has indicated that lncRNA plays an important role in the pathogenesis of certain inflammatory diseases [48–56]. In this study, therefore, we investigated the role of a long non-coding RNA, LINC00926, in the regulation of inflammatory gene expression in PTSD. To that end, we focused our studies on inflammatory genes such as IFNG and IL17A, which were upregulated in patients with PTSD [16, 17]. Additionally, recent studies from our lab also showed that WNT signaling pathway which regulates inflammation was significantly upregulated in PBMCs due to PTSD when compared to normal controls [30].

WNT signaling dysregulation has been reported in several types of cancers [57–60]. It is becoming increasingly evident that WNT signaling might be dysregulated in other inflammatory diseases as well. For example, WNT5A has been reported to be upregulated in macrophages after exposure to pathogens and also during differentiation of monocytes to dendritic cells after exposure to granulocyte macrophage colony-stimulating factor and IL4 [61]. In another report it was shown that dental pulp cells have elevated expression of WNT5A indicating its role in this inflammatory disease [62]. It was also reported that WNT5A is overexpressed in the atherosclerotic lesions from both murine and human specimens [63]. Together, such reports suggest that WNT signaling pathway could be involved in positively influencing inflammatory responses. However, the mechanism by which the expression of WNT signaling pathway molecules, including WNT ligands, is regulated by epigenetic modulations is not well understood. Moreover, regulation of inflammation by WNT signaling in PTSD has not been reported before. Because the presence of H3K4me3 upregulates the nearby gene, in agreement, we saw higher transcript levels of WNT10B in PTSD patients. These observations are suggestive of a role of H3K4me3, which is a positive regulator of gene expression. As immune system genes have been reported previously to be regulated by epigenetic mechanisms [15–18, 47, 64, 65], one can conclude from our present observations that upregulated expression of WNT10B in PTSD patients is an outcome of higher H3K4me3 around its promotor.

It has been shown that lncRNAs can mediate the writing or erasing of epigenetic marks like histone modifications around the promotor of target genes. To bring about this effect, the lncRNA associates physically with histone modifying enzymes, bring them to the promotor of the target gene and mediates modification of the histone proteins, thereby resulting in the regulation of that gene. In agreement with the above-described mechanism, upon co-immunoprecipitation with anti-MLL1, we saw significant enrichment of LINC00926. Thus, these data implied that the molecules were physically attached to each other. Therefore, as seen with lncRNAs HOTAIR and XIST in mediating histone modifications, we believe that LINC00926 is involved in bringing MLL1 to the WNT10B promotor and assist in the introduction of H3K4me3 around its promotor in PTSD patients. This notion is further supported by our observations where WNT10B expression is decreased upon knocking down the LINC00926. Furthermore, regulation of immune system genes by histone marks has been previously reported by several groups, as mentioned earlier. Thus, we also believe that our observation is significant and provide a new approach to look at how immune system genes are regulated in PTSD patients.

WNT10B promotor engagement by the histone methyltransferase-LINC00926 complex was a prerequisite for histone modification to occur. Significant enrichment of WNT10B promotor region after ChIP-quantitative real time PCR, as observed by us, is a clear indication of this phenomenon. Because our ChIP-seq data showed higher H3K4me3 around upstream of WNT10B TSS and corresponding to this, the enriched region of the promotor was upstream of the WNT10B promotor, we believe that the engagement of the WNT10B promotor is apparent in order to regulate the expression of WNT10B. This observation strongly suggests that the MLL1-LINC00926 complex is attached with the WNT10B promotor at the upstream region and because the presence of H3K4me3 in the promotor is associated with activation of a gene, these data further support that MLL1-LINC00926 complex is present before the TSS of WNT10B and introduce H3K4me3. Consequently, if MLL1-LINC00926 mediated phenomenon is responsible for the introduction of H3K4me3, alteration in the level of either of these molecules should affect the expression of WNT10B. To that end, when MLL1 (H3K4me3 writer), KDM5B (H3K4me3 eraser) or LINC00926 was knocked down, we saw alterations in the levels of WNT10B. Thus, these observations clearly indicated that WNT10B expression is regulated by H3K4me3.

The additional question we tried to address was whether elevated expression of WNT10B favors the increased expression of inflammatory cytokines. It has been reported that elevated expression of molecules of WNT signaling pathway, particularly WNT5A and WNT3A, as described earlier, is associated with inflammation but it is not known whether WNT10B also favors similar outcomes. Our data suggested that WNT10B increased the expression of IFNG and IL17A. This observation would mean that if the immune cells in PTSD patients are already in an activated state, presence of WNT10B could push that to a higher level of expression and contribute more to the inflammatory state. Consistent with this observation, either blocking of WNT signaling by an inhibitor or knocking down of WNT10B or LINC00926 by siRNA should lead to decreased IFNG and IL17A, as was seen in our study. From these data, one can conclude that signaling through WNT10B promotes elevated expression of inflammatory cytokines IFNG and IL17A, and this can contribute to the inflammatory state in PTSD patients.

Our report is the first to show that WNT signaling pathway, a pathway involved in various critical fundamental physiological functions like cell fate decision, development, differentiation and many others, is dysregulated in PTSD and is involved in the elevated expression of pro-inflammatory genes. LINC00926 mediated H3K4me3 in the promotor of WNT10B contributes to the enhanced WNT signaling. However, it is unclear whether an increased WNT10B expression in PBMCs leads to further inflammation in CNS in PTSD patients, or the stress in PTSD causes enhanced LINC00926 expression and WNT signaling in PBMCs. If the latter is the case, one would expect LINC00926 could contribute to comorbidities associated with PTSD. Another limitation of this study is that nearly 80% of PTSD patients have a comorbid disorder. The common comorbidities including depression, anxiety, bipolar disorder, alcohol, and substance abuse, eating disorder, and it is known that epigenetic regulation plays important roles in these disorders [66–68]. It is possible that the epigenetic regulation including LINC00926 mediated WNT signaling PTSD could be due to comorbidities. Nevertheless, this study suggests that LINC00926 could be a potential biomarker and therapeutic target for PTSD.
